# Retrospective Cohort Analysis of Outpatient Antibiotic Use for *Clostridioides difficile*-Indicated Agents in British Columbia, from 2000 to 2018

**DOI:** 10.1155/2023/9465158

**Published:** 2023-02-10

**Authors:** Ariana Saatchi, Sungeun Kim, Fawziah Marra

**Affiliations:** Faculty of Pharmaceutical Sciences, University of British Columbia, Vancouver, BC, Canada

## Abstract

**Background:**

*Clostridioides difficile* (CDI) is the most common cause of nosocomial diarrheal infections. Historically, metronidazole was the first-line treatment, but guidelines now indicate oral vancomycin and fidaxomicin as primary antibiotics for initial episodes. A provincial stewardship program has operated in British Columbia (BC), since 2005. Since the program's inception, surveillance of antibiotic use has been ongoing. However, this is the first study to review community-acquired CDI-indicated antibiotic use. Moreover, this study offers the first interpretation of fidaxomicin use in BC since its addition to the provincial formulary.

**Methods:**

A retrospective cohort analysis included all outpatient dispensations for CDI-related antibiotics from January 1, 2000, to December 31, 2018. Antibiotic dispensations were extracted for metronidazole, vancomycin, and fidaxomicin. Consumption rates were calculated as prescriptions per 1000 population. Rates were examined overall and then stratified by medication, age, and sex. Secondary outcomes of interest included an examination of adherence to provincial special authority criteria; and proportions of outpatient antibiotic use attributable to administrative health records for CDI.

**Results:**

The average annual rate of prescribing was 18.5 per 1000 population for all CDI-indicated antibiotics. The rate of prescribing increased (15%) over the 19-year study period, from 17.2 to 19.8 dispensations per 1000 population. Metronidazole accounted for the most antibiotics dispensed in every study year; however, by 2018 it demonstrated the most modest increase in use (15%). In comparison, fidaxomicin increased by 226% by 2018. Vancomycin had the highest percentage increase (621%), with the greatest change occurring from 2014 to 2015, correlating to the dissemination of new clinical practice guidelines.

**Conclusion:**

This is the first study to evaluate outpatient prescribing for CDI-indicated antibiotics, and one of the few studies to examine fidaxomicin since its introduction to Canadian formularies. Although causation cannot be inferred from study results, oral vancomycin, and fidaxomicin use has increased in line with, or in advance-of guidelines.

## 1. Introduction

*Clostridioides difficile* (formerly *Clostridium difficile*) is the most common cause of hospital-associated diarrheal infections [1]. Pathogenic strains produce toxin *A*, *B*, or a binary toxin upon colonization of the gut, which can lead to a range of symptoms from diarrhea, colitis, and in severe cases death [2]. *Clostridioides difficile* infection (CDI) has traditionally been characterized as nosocomial, or healthcare-associated (HA), with the incidence of HA-CDI being 2.1/10 000 to 6.5/10 000 inpatient days [3]. However, mounting evidence shows that cases of community-associated CDI (CA-CDI) are increasing [4, 5]. A case of CA-CDI is defined as either symptom onset in the community or outpatient setting; symptom development at least 12 weeks following a hospital discharge; or symptom onset within 48 hours after admission to a healthcare facility [1]. International data for CDI reports approximately 20–27% of CDI cases are community-acquired, with an annual incidence of 20–30 cases of CA-CDI per 100,000 population [5]. In British Columbia (BC), the incidence of CA-CDI has increased from 16.74 cases per 100 000 population in 2011–2012 to 17.78 cases per 100 000 population by 2016–2017 [3].

Treatment for CDI centers around antibiotic therapy [6, 7]. Historically, metronidazole was the first-line antibiotic treatment for CDI, but updates to IDSA guidelines now indicate oral vancomycin and fidaxomicin as first-line agents, displacing metronidazole as the primary antibiotic for an initial episode of CDI [6]. Both oral vancomycin and fidaxomicin have been found to be superior to metronidazole against the initial episode, first recurrence, and nonsevere CDI; with similar treatment outcomes for both fidaxomicin and oral vancomycin in the treatment of severe cases [8]. In comparison to vancomycin, fidaxomicin demonstrates better sustained bactericidal rates [9]. However, oral vancomycin is often still prescribed over fidaxomicin as the latter is up to 14 times more expensive, and often not included within regional formularies for coverage [10]. Beyond bactericidal efficacy, treatment with fidaxomicin and/or vancomycin results in lower rates of CDI shedding and environmental contamination, when compared to metronidazole [11].

A community-based antimicrobial stewardship program has operated in the province of BC, since 2005. The DoBugsNeedDrugs™ program educates the public on judicious antibiotic use, while also disseminating clinical resources to healthcare professionals to promote the principles of antimicrobial stewardship and support patient care. Since the program's inception, provincial surveillance of antibiotic use has been ongoing. However, this is the first study to review CA-CDI-indicated antibiotic use, and moreover, this study offers the first interpretation of fidaxomicin use in BC since its addition to the provincial formulary. The objective of this study is to examine provincial trends in antibiotic dispensing for metronidazole, vancomycin, and fidaxomicin, from 2000 and 2018.

## 2. Methods

### 2.1. Data Sources

Canadian citizens and permanent residents receive health coverage through provincial health insurance. As such, the Ministry of Health in BC maintains several health data indices, which contain comprehensive medical information for the population of BC. Community prescription data is contained within the BC PharmaNet, a centralized system linking outpatient pharmacies and dispensations [12]. All antimicrobials were recorded in this system except those used for the treatment of sexually transmitted infections and HIV. Medications dispensed within a hospital, or inpatient care setting, are also not available in the BC PharmaNet. The medical services plan (MSP) billing system contains records for outpatient care covered by the provincial, universal insurance program [13]. All claims submitted by physicians are available, including diagnostic codes. Hospitalization data are available through the discharge abstracts database (DAD), recording all diagnostic information for provincial acute care hospitals [14]. Inpatient antibiotic data is not available through DAD. Patient demographics (e.g., age, sex) were provided through a consolidation file [15]. Access to the data provided by the data stewards is subject to approval, but can be requested for research projects through the data stewards or their designated service providers. All inferences, opinions, and conclusions drawn in this publication are those of the authors and do not reflect the opinions or policies of the data stewards.

### 2.2. Study Population

Our study included all residents of BC, from January 1, 2000, to December 31, 2018, that were dispensed a prescription for a CDI-related antibiotic. Antibiotics were classified based on the Anatomical Therapeutic Chemical (ATC) classification system developed by the World Health Organization. Antibiotic dispensations were extracted from PharmaNet, and the following three agents were included as follows: metronidazole (J01XD01), vancomycin (J01XA01), and fidaxomicin (A07AA12). Antimicrobials were limited to oral use only. Multiple prescriptions per subject were permitted in our analyses. All cells with *n* < 6 were excluded from subsequent analyses to preserve subject anonymity.

### 2.3. Primary Outcomes & Statistical Analyses

Primary outcomes included the rates of antibiotic use. Consumption rates were calculated as prescriptions per 1000 population per year, using age- and gender-specific denominator estimates, available for the population through statistics BC. Rates of antibiotic use were examined overall and then stratified by the three clinically relevant, CA-CDI-related drugs (i.e., metronidazole, vancomycin, and fidaxomicin). For each medication, rates of use were further examined by sex and age category. Age categories included pediatrics (aged <19 years), adults (aged 19–64 years), and seniors (aged >65 years). Change over time was calculated for all rates, comparing the percentage change between the first and final, study years.

### 2.4. Secondary Outcomes

Although oral vancomycin and fidaxomicin are exclusively used for the treatment of CDI, metronidazole is used as an antiprotozoal agent for a variety of indications. Secondary outcomes examined the proportions of antibiotic use attributable to community and/or hospital physician records for CDI. CA-CDI cases were identified using ICD-9 code “008” in MSP, while HA-CDI cases utilized ICD-10 code “A04.7” through DAD. A prescription and diagnosis were linked through an algorithm that tied the dispensation date to a physician visit within a 5-day lookback period. Prescriptions that did not match a CDI physician record were categorized as “unlinked.”

Metronidazole has been included within the BC formulary since 2001, with vancomycin being covered since 2004, and fidaxomicin was added in 2014. However, both vancomycin and fidaxomicin receive coverage under “special authority” in BC, with requirements that must be met prior to patient eligibility, and reimbursement. In order to receive provincial coverage, a prescription of oral vancomycin requires the following: (1) a preceding course of metronidazole (excluding documented allergy/intolerance); (2) patient symptoms of moderate to severe disease; (3) patient experiencing a second disease recurrence; or (4) patient initiated on vancomycin as an inpatient. For fidaxomicin, either a documented allergy/intolerance to vancomycin; or an unsuccessful preceding course of oral vancomycin coupled with a high-risk hospitalization is required to achieve “special authority” coverage. Both oral vancomycin and fidaxomicin were examined to see what proportion of dispensations upheld the BC coverage criteria, respectively. A 14-day lookback period was utilized in linking prescriptions in order to allow a complete first prescription course, secondary physician visit, and subsequent dispensation.

## 3. Results

An average of 61 809 unique patients were prescribed an antibiotic indicated for CDI in any given year, with over 1.5 million antibiotic dispensations between 2000 and 2018 (Table 1). During this period, the population of BC increased by 24% to 5 001 170 in 2018. Regarding patient demographics, the mean age of the cohort increased from 46 to 50 years of age by 2018. The average patients were adult females living in urban regions. No differences were observed based on income quintiles.

### 3.1. Overall Rate of Prescribing for CDI-Indicated Antibiotics

**Figure 1:**
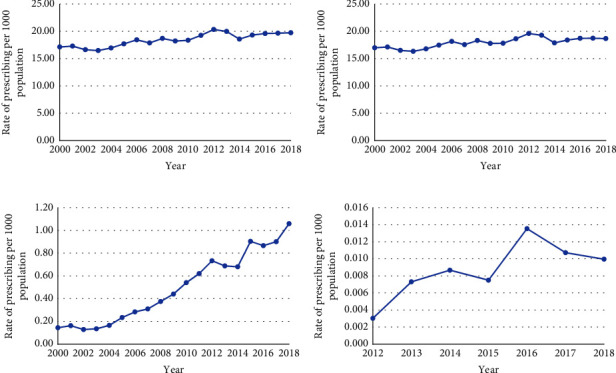
Overall rate of total oral CDI-indicated antibiotic prescribing and by individual antibiotic in British Columbia, 2000–18: (a) shows the rate of total antibiotic prescribing, combining all three antibiotics of interest; (b–d) show the rate of prescribing for metronidazole, vancomycin, and fidaxomicin, respectively, throughout the study period.

Overall, an average annual rate of 18.5 antibiotic dispensations per 1000 population was observed for all antibiotics. The rate of prescribing increased (15%) over the 19-year study period, from 17.2 to 19.8 dispensations per 1000 population (Figure 1(a)).

**Figure 2:**
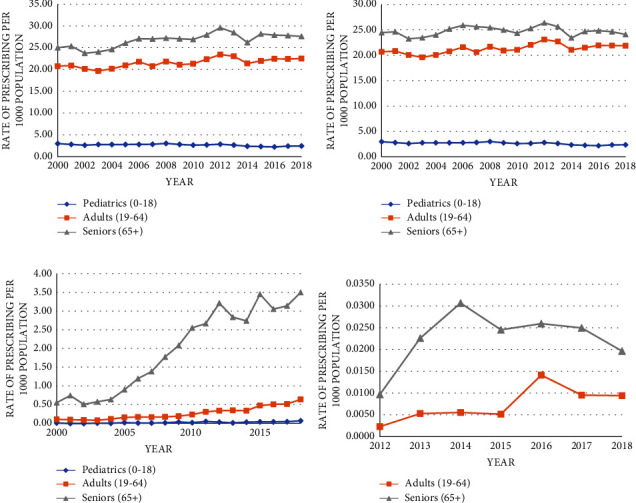
Rate of total oral CDI-indicated antibiotic prescribing and by individual antibiotic, stratified by age categories (pediatric: 0–18; adult: 19–64; senior: >65), in British Columbia, 2000–18: (a) shows the rate of total antibiotic prescribing for each age group; (b–d) show the rate of rate prescribing for metronidazole, vancomycin, and fidaxomicin, respectively, for each age group.

**Figure 3:**
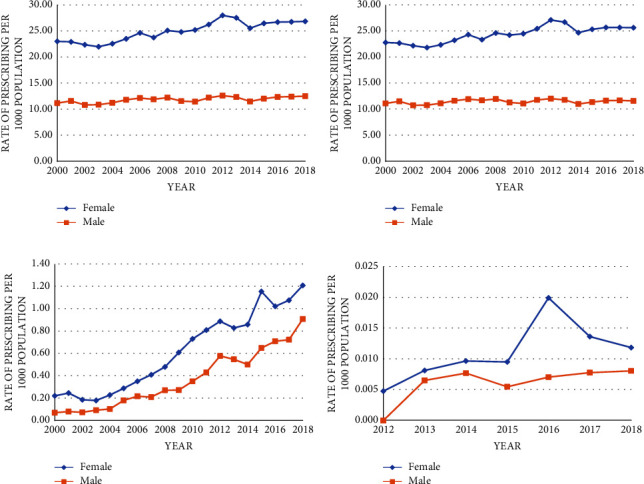
Rate of total oral CDI-indicated antibiotic prescribing and by individual antibiotic, stratified by sex, in British Columbia, 2000–18: (a) shows the rate of total antibiotic prescribing for each sex; (b–d) show the rate of rate prescribing for metronidazole, vancomycin, and fidaxomicin, respectively, for each sex.

By medication of interest, all three drugs increased in use over the study period, but by varying magnitude (Figures 1(b)–1(d)). Figures 2 and 3 show the overall rate of prescribing for each antibiotic, as well as rates based on sex and age, between 2000 and 2018. Metronidazole accounted for the most antibiotics dispensed in every study year; however, by 2018, it demonstrated the most modest increase (15%) in use (17 to 18.6 prescriptions per 1000 population). Vancomycin had the highest percentage increase of 621% (0.15 to 1.06 prescriptions per 1000 population by 2018), with the greatest change occurring between 2014 and 2015, with a jump in the rate from 0.68 to 0.91 prescriptions per 1000 population; the highest rate was achieved in 2018 (1.06 prescriptions per 1000 population). Fidaxomicin increased by 226% (0.003 to 0.010 prescriptions per 1000 population by 2018), reaching its highest rate in 2016 (0.014 prescriptions per 1000 population), and achieving the greatest change between 2015 and 2016 (0.008 to 0.014 prescriptions per 1000 population).

On average, the rate of prescribing per 1000 population for fidaxomicin was 0.009 from 2012 to 2018 (data were available later than the other two antibiotics), compared to 18 for metronidazole and 0.5 for vancomycin from 2000 to 2018. Additionally, the rate for fidaxomicin reached a peak of 0.014 prescriptions per 1000 population in 2016 before decreasing to 0.010 prescriptions per 1000 population by 2018.

When observing trends based on age categories, seniors were generally prescribed CDI-related antibiotics at a higher rate than children and adults (Figures 2(a)–2(d)). This trend was consistent among all three antibiotics and across the study period. Vancomycin, in particular, had the greatest increase in the rate of prescribing for seniors (528%) over adults (480%), and children (374%) (Figure 2(c)). Fidaxomicin prescribing followed the same trend, but the percentage increase was higher for adults (299%) compared to seniors (10%); no pediatric dispensations of fidaxomicin were identified (Figure 2(d)). Metronidazole prescribing saw a decrease in pediatrics (20%) and seniors (2%), while adults increased by approximately 6% (Figure 2(b)). However, the general trend in prescribing remained consistent in that seniors were prescribed the antibiotic at a higher rate than the other two age categories.

Sex stratification showed a consistent pattern in prescribing (Figure 3(a)). Females were prescribed antibiotics at a greater rate compared to males for all three antibiotics. However, vancomycin and fidaxomicin prescribing rates for males and females showed a stronger positive association compared to the trend observed for metronidazole (Figures 3(b)–3(d)).

### 3.2. Sensitivity Analysis

A total of 0.37% of metronidazole prescriptions, 6% of vancomycin, and 18% of fidaxomicin were successfully linked to an outpatient physician record for CA-CDI. Similarly, 0.18% of metronidazole prescriptions, 3% of vancomycin, and 5% of fidaxomicin were successfully linked to an inpatient record for HA-CDI (Table 2). Across the study period, 14% of vancomycin prescriptions were linked to prior use of metronidazole. In comparison, 34% of fidaxomicin prescriptions were linked to prior use of vancomycin.

## 4. Discussion

This is the first study to evaluate outpatient prescribing for CDI-indicated antibiotics, and one of the few studies to evaluate fidaxomicin prescribing since its introduction to the Canadian marketplace. We observed a 15% increase in total provincial antibiotics over the 19-year study period. A positive overall trend was reported for each antibiotic but was most pronounced for oral vancomycin (621%), followed by fidaxomicin (226%), with metronidazole demonstrating the most modest trend (15%) over the 19-year study period. Although stagnant in use when compared to the other medications, metronidazole was dispensed at rates far and above vancomycin and/or fidaxomicin across all study years. These results are corroborated by previous provincial analyses, which report a 97% total increase in prescribing for the J01X ATC class of “other antimicrobials”; comprised of metronidazole, vancomycin, and more [16].

Updates to clinical practice guidelines over time also correspond to changes observed in prescribing rates for the three antibiotics. During the early 2000s, the first-line antimicrobial therapy for CDI was metronidazole, which remained the guideline-recommended first-line agent for CDI until 2012, with preferred use for initial mild episodes of CDI. At this time, vancomycin was reserved for use in severe episodes, due to the adequate efficacy of metronidazole for CDI and the risk of vancomycin-resistant enterococci acquisition in hospitals [17–23]. However, the study results show an exponential increase in vancomycin prescribing beginning in 2004, 6 years prior to the IDSA guideline updates in 2012 (Figure 1(c)). Prior to 2004, the rate of prescription for vancomycin was relatively stable (0.2 prescriptions per 1000 population). This increase may be attributable to a change in disease severity secondary to the emergence of a hypervirulent strain in Canada and the United States in 2003 [23–26]. This new strain, the North American pulsed-field type 1 (NAP1/B1/027), is associated with greater production of toxins compared to control strains [23]. As vancomycin was recommended for the treatment of severe illness, the shift in bacterial strain may account for the increased physician preference during this period [21, 27, 28]. Vancomycin use increased further after the guideline change in 2012, which recommended it as the drug of choice for most cases of CDI, unless very mild and uncomplicated.

A growing body of literature supports the superiority of fidaxomicin, when compared to both metronidazole and oral vancomycin, for the treatment of CDI and for less patient sequelae [8, 29–32]. This study found that the first outpatient prescriptions of fidaxomicin were dispensed in 2012, two years prior to formulary incorporation. Despite a steady upwards trend in use, prescribing rates for fidaxomicin remain low. A special authority status, elevated cost of treatment, and a judicious provincial stewardship culture could each influence the ongoing reservation of fidaxomicin use in outpatient care. No pediatric use of fidaxomicin was identified throughout the study period; however, this result was expected as the use of this medication remains limited to individuals aged ≥18 years [33]. Overall, the trends of antibiotic use reported in this study reflect updates made to clinical practice guidelines throughout the study period. The most recent IDSA guidelines became available in 2018, reiterating the position of vancomycin and fidaxomicin as first-line agents, and correlating to the continued increases in the use of both drugs in later study years. Future research efforts should continue to monitor these CDI-indicated drugs in order to monitor guideline adherence. As previous antibiotic use remains a leading risk factor for CDI, particularly fluoroquinolone exposure, it is notable that provincial quinolone use declined by 22%, over the same study period [6, 16]. However, provincial rates of CDI continue to trend upward, particularly within the community [3–5].

The average patient was approximately 48 years of age (Table 1). Previous studies have identified that CA-CDI patients are typically younger than those with HA-CDI, with the median age of CA-CDI patients being 50 years, in comparison to 72 years for HA-CDI [34]. In another study from the US, Chitnis et al. also reported a median age of 51 years for confirmed CA-CDI patients [35]. The age-based trends reported for CDI-indicated antibiotics are supported by the literature surrounding patient cohorts and associated ages.

The sensitivity analyses of vancomycin and fidaxomicin found that only 14% of vancomycin prescriptions were linked to a preceding metronidazole dispensation, while 34% of fidaxomicin prescriptions were linked to a prior vancomycin dispensation. These percentages were lower than expected, especially given the criteria necessary to achieve “special authority” status include an ineffective but adequate preceding course of treatment for each medication. With respect to vancomycin, a lower linkage may have been achieved as some patients may not have been prescribed metronidazole within the permissible 14-day lookback period. Other conditions for oral vancomycin under “special authority” include moderate to severe CDI; recurrence, or continuation of therapy following inpatient care [36]. As these additional criteria do not specify the prior dispensation of metronidazole, the above scenarios are likely hypotheses underlying “unlinked” oral vancomycin use. Similarly, for fidaxomicin (34% linkage), further “special authority” criteria dictate its use if it is prescribed by an infectious disease physician or gastroenterologist; documented allergy/intolerance for vancomycin, or severe illness and risk of hospitalization [37]. Moreover, there may be some scenarios in which a 14-day lookback window is insufficient to best provide an accurate linkage of fidaxomicin to a prior vancomycin dispensation. This study offers the first high-level interpretations of preceding medication use for CDI-related “special authority” antibiotics. However, further research is required to properly elucidate the provincial “special authority” criteria, subsequent adherence, and proportions attributable to each of the aforementioned criteria.

Overall, only 0.53% of the total antibiotics were linked back to an outpatient CDI-related physician record, with 0.27% linked to an inpatient record. Fidaxomicin reported the highest proportions of linkage across both healthcare settings (Table 2). Given the wide-ranging indications for metronidazole use (e.g., reproductive system, skin and soft tissue, and sexually transmitted infections), the rates reported are not surprising. The iteration of clinical practice guidelines which decentred metronidazole as a first-line agent and the introduction of superior treatment options for CDI are also potential explanations. However, the low linkage of vancomycin and/or fidaxomicin, which are primarily indicated for CDI, highlights the need for further research into our provincial administrative billing codes for accuracy. Moreover, it is integral to understand which billing codes are being utilized for CA-CDI cases in order to identify cases of infection within our provincial data.

As a retrospective study using administrative health data, there are limitations to consider. Unfilled prescriptions would not be identified within dispensation records, as such our discussion of antibiotic prescribing may be an underrepresentation of provincial use. Moreover, the absence of accurate culture, lab, and allergy data limited our characterization of “special authority” use to preceding antibiotic dispensation records only. Furthermore, our use of physician billing codes (ICD-9/10) may be subject to misclassification bias, as CDI case identification was reliant on accurate coding by physicians. It is notable that Canadian primary care physicians' claim data have a high positive predictive value for the diagnosis of common infections [38]. However, this may not be a valid extrapolation to CDI. Finally, annual cases of CDI were not identified within this study, as accurate case identification of CA-CDI, is not yet been validated using outpatient administrative health data. Antibiotics indicated for the treatment of CA-CDI were reported and examined for changes over time relative to CDI clinical practice guidelines.

## 5. Conclusion

CDI cases in outpatient care continue to rise, as the infection has expanded beyond nosocomial and into community circulation. In tandem, since 2000, this study reports an overall increase in the outpatient dispensation of CDI-indicated antibiotics. Although causation cannot be inferred from study results, oral vancomycin and fidaxomicin use has increased over the study period, in line with, or in advance of guideline changes made throughout the 19-year study period. Furthermore, research is necessary to elucidate adherence to provincial “special authority” criteria in relation to both medications. Moreover, antibiotics dispensed in the community are typically issued to a younger cohort than those reported in HA-CDI cases, highlighting the importance of reframing CDI, and associated antibiotic use as a widespread outpatient concern rather than a nosocomial risk for seniors.

## Figures and Tables

**Table 1:** Cohort characteristics, overall data from 2000 to 2018.

Cohort characteristics	Overall (2000–18)
Total number of unique patients	1 174 374
Average patients per year	61 809
Age	
0–18	38 446
19–64	897 639
65+	238 289
Mean age	48
Sex	
Female	808 309
Male	365 952
Income quintile	
Quintile 1 (lowest)	259 536
Quintile 2	234 994
Quintile 3	224 572
Quintile 4	218 377
Quintile 5 (highest)	203 054
Missing	23 337
Rural/urban status	
Rural	226 049
Urban	901 790
Missing	46 535
Total indicated antibiotics prescribed	
Metronidazole	1 523 031
Vancomycin	43 540
Fidaxomicin	294

**Table 2:** Percentage of CDI-indicated antibiotics linked to the outpatient (ICD-9 diagnostic code “008”) database and the discharge (hospital) database (ICD-10 diagnostic code “A04.7”) throughout the entire study period, 2000–18.

Type of antibiotic	Count linked to “008”	Percentage linked (%)	Count linked to “A04.7”	Percentage linked (%)
Metronidazole	5565	0.37	2768	0.18
Vancomycin	2633	6.05	1386	3.18
Fidaxomicin	53	18.03	15	5.10
Overall	8251	0.53	4169	0.27

## Data Availability

The underlying data for this research is protected by the BC Ministry of Health and cannot be made publicly available.
